# Is Hepatitis E Virus a Neglected or Emerging Pathogen in Egypt?

**DOI:** 10.3390/pathogens11111337

**Published:** 2022-11-12

**Authors:** Ibrahim M. Sayed, Sayed F. Abdelwahab

**Affiliations:** 1 Department of Biomedical and Nutritional Sciences, University of Massachusetts Lowell, Lowell, MA 01854, USA; 2 Department of Medical Microbiology and Immunology, Faculty of Medicine, Assiut University, Assiut 71515, Egypt; 3 Department of Pharmaceutics and Industrial Pharmacy, College of Pharmacy, Taif University, P.O. Box 11099, Taif 21944, Saudi Arabia

**Keywords:** HEV, zoonoses, water, neglected, emerging pathogen, Egypt

## Abstract

Though Egypt ranks among the top countries for viral hepatitis and death-related liver disease, Hepatitis E virus (HEV) is a neglected pathogen. Living in villages and rural communities with low sanitation, use of underground well water and contact with animals are the main risk factors for HEV infection. Domestic animals, especially ruminants and their edible products, are one source of infection. Contamination of water by either human or animal stools is the main route of infection. In addition, HEV either alone or in coinfection with other hepatotropic viruses has been recorded in Egyptian blood donors. HEV seropositivity among Egyptian villagers was 60–80%, especially in the first decade of life. Though HEV seropositivity is the highest among Egyptians, HEV infection is not routinely diagnosed in Egyptian hospitals. The initial manifestations of HEV among Egyptians is a subclinical infection, although progression to fulminant hepatic failure has been recorded. With the improvement in serological and molecular approaches and increasing research on HEV, it is becoming clear that HEV represents a threat for Egyptians and preventive measures should be considered to reduce the infection rate and possible complications

## 1. Introduction

Liver disease is one of the most common causes of death for Egyptians [1]. Among reported liver disease, viral hepatitis causes disease in 10% of the population (around 8–10 million) and millions of Egyptians are at risk of acquiring liver diseases [2]. However, not all the known viral hepatitis causes are diagnosed in Egypt. Hepatitis E virus (HEV) is one of the undiagnosed viruses in Egypt. HEV is an RNA virus which causes acute viral hepatitis [3]. HEV belongs to family *Hepeviridae*, which is divided into two subfamilies; *Orthohepevirinae* and *Parahepevirinae* [4]. The *Parahepevirinae* subfamily include cutthroat trout virus (*Piscihepevirus heenan*)[4]. The *Orthohepevirinae* subfamily includes four genera; *Paslahepevirus, Rocahepevirus, Chirohepevirus* (bat) and *Avihepevirus* (birds) [4]. *Paslahepevirus* includes human and mammalian HEV isolates which are categorized into at least eight genotypes (HEV1-8) [4], of which five were reported with human infections (HEV1-4 and HEV-7) [5,6]. HEV-1 and HEV-2 are reported mainly in developing countries and they are associated with outbreaks and waterborne HEV infection [7]. HEV-3 and HEV-4 are associated with foodborne HEV infections and they are common in industrialized countries [8]. HEV-7 was detected in camels in the Middle East [9,10,11,12,13]. Dromedary camel anti-HEV IgG and HEV RNA were detected in domestic and imported camels in Saudi Arabia, suggesting that camels could be a potential source of zoonotic HEV infection there [10,11]. Similarly, HEV-7 was detected in dromedary camels in Israel and high HEV seroprevalence was also recorded among residents there [12,13]. HEV-8 was reported in Bactrian camels, which was infectious to non-human primates and rabbits, but not humans until now [14,15,16]. HEV-1 and HEV-3 isolates were reported in Egypt [17,18,19]. Limited sequence data for HEV isolates were identified in Egypt and most HEV genotype 1 isolates were categorized into subtypes 1a, 1b, 1e and 1f [17,18,20,21,22]. *Rocahepevirus ratti* incudes rat-derived HEV isolates which were recently reported with human infections [4,23,24].

HEV replicates in the liver causing acute hepatitis with symptoms similar to other causes of hepatitis. Some HEV infections are subclinical in nature, while others are severe and could progress to hepatic failure [25]. HEV-1 and HEV-2 infections cause severe outcomes during the third trimester of pregnancy, with a mortality rate of 20% [26,27,28]. Age, liver disease and coinfection with other viruses are other risk factors for the complications observed during HEV infection [29,30,31,32,33]. A recent study reported that a very low HEV load, missed from blood donor screening in England, can cause severe and fulminant hepatitis among recipients [34]. Chronicity is observed in immunocompromised patients, especially with HEV-3 and HEV-4 [35,36]. Extrahepatic manifestations were documented with HEV-3 and HEV-4 infections which mainly affect the renal and nervous systems [37].

Animals play an important role in HEV transmission in developed countries. Pigs, wild boar, rabbits, ruminants, deer and others are HEV reservoirs and sources of infection, either via frequent contact and exposure or ingestion of their contaminated products [38,39,40,41]. In Egypt, though limited data are available about zoonotic HEV transmission, some studies showed that ruminants and their products could be a potential source of infection [42,43].

The HEV genome includes three main open reading frames (ORF). ORF1 mediates viral replication through the encoded enzymes, RNA dependent RNA polymerase, helicase, and cysteine protease [44]. ORF2 assembles the viral capsid [45,46] and ORF3 forms a small phosphoprotein required for HEV release [47,48]. A cap is present at the 5′ of the genome which is required for viral infectivity [49]. ORF2 has several forms, which are either infectious (non-glycosylated) or non-infectious (glycosylated forms) [45,46].

HEV research has been improved due to the recent advances in molecular and serological developments in in vitro and in vivo systems for studying and diagnosing HEV infection [50,51,52].

In Egypt, earlier studies linked HEV infection to low sanitation in villages and rural communities. Consumption of water from a non-cleaned source such as underground wells, ground or public water were the main risk factors [53,54]. Most seropositive Egyptians were asymptomatic and 60–80% of were positive during the early decade of life [43,55]. Although a high seropositivity for HEV antibodies was reported among Egyptians, the virus is neglected and not routinely diagnosed. Previously, most HEV seropositive subjects and HEV-infected patients were either asymptomatic or developed a mild self-limiting course [18,43,53,54]. However, recent studies showed that HEV was the cause of fatal fulminant hepatitis among Egyptians [21,56], with a high prevalence rate among blood donors with other endemic hepatotropic pathogens such as hepatitis B virus (HBV) and hepatitis C virus (HCV) [57]. Taking into consideration the high rate of viral hepatitis and death associated with liver diseases in Egypt, we assume that this pathogen is responsible for several infections and deaths among Egyptians.

This review gives updates on the status of HEV infection in Egypt, highlights the possible sources of infections (Zoonoses and water) among Egyptians and emphasizes the importance of HEV detection and diagnosis whether in the environment, animals, or hospitals, in order to reduce the risk of complications associated with this pathogen.

## 2. Zoonotic HEV Infection in Egypt

Several studies confirmed that the main risk factor for HEV infection and/or acquiring HEV antibodies was living in villages and rural communities. Reports in different Egyptian governorates examined the prevalence and risk factors for HEV infection among Egyptians (Table 1). In these regions, animals are living and defecating close to humans and the stool could be the source of water contamination in these villages. Therefore, infection can spread to many villagers. This explanation could be the reason for higher anti-HEV seropositivity among villagers. Besides, susceptibility of domestic animals to HEV has been confirmed in these regions. In the Middle East, camels are confirmed sources for HEV infection through ingestion of milk and meat from seropositive animals. Highly HEV seropositive camels, either domestic or imported, were reported [10,11,12,13]. Although camels are abundant in Egypt, there are no reports on the association of HEV infections among Egyptians and camels.

Below, we describe the animals reported as potential sources of HEV infection among Egyptians.

### 2.1. Ruminants

#### 2.1.1. Cows

At first sight, cows were considered as accidental hosts, not actual reservoirs for HEV. However, subsequent studies confirmed that cows and their products are potential sources for zoonotic HEV infection in several countries such as China, Turkey, Egypt, Central Africa, and Jordan [70,71,72,73,74,75]. In the previous studies either anti-HEV antibodies, HEV Ag and/or HEV RNA were detected in the plasma, stool, liver and/or milk of the cows [70,71,72,73,74,75]. HEV excreted in cow milk was infectious in vivo to animal models [74,76]. In Egypt, there are two reports of HEV infection in cows. El-Tras et al. reported that cows were the most susceptible animals among Egyptian ruminants to HEV. About 21% of the tested cows were seropositive and male cows were significantly more susceptible than female [42]. The second report was made by Sayed and colleagues, the authors assessed HEV prevalence in cow milk samples collected from 12 farms from villages in Assiut Governate [72]. Two hundred and twenty samples representing samples collected from 11 farms were HEV negative. Some milk samples from a farm (in villages of Abnub city) were positive for HEV markers. In the first collection, eight milk samples were positive for anti-HEV IgG and 10 samples were positive for anti-HEV IgG in the second collection from the same animals a month later [72]. The level of anti-HEV antibodies and the number of positive animals were higher in the second collection than in the first collection, indicating the circulation of HEV among these animals [72]. Interestingly, HEV RNA and HEV Ag were detected in one animal [72]. The isolated virus belonged to HEV subgenotype 3a [72].

#### 2.1.2. Buffalos

Buffalos were reported as a new HEV reservoir in China [77]. HEV RNA was recorded in 4.72% of buffalos’ serum samples and 7.5% of milk samples of these animals [77]. The isolated viruses belonged to HEV genotype 4. None of the tested buffalos were positive for anti-HEV IgG, while one animal tested positive for anti-HEV IgM [77]. Anti- HEV IgG was detected in one out of five buffalos (20%) present in the rural settings of the Lao People’s Democratic Republic [78]. Villagers who were in contact with ruminants in this area had higher HEV seropositivity compared to control groups [78]. Buffalos are common in Egyptian rural communities and villages. They are common food animals for Egyptians. El-Tras et al. assessed the seroprevalence of HEV among humans and ruminants including buffalos in the KafrElsheikh governorate, Nile Delta [42]. Animal samples were collected from the area including seropositive humans. Anti-HEV IgG was detected in 14% of buffaloes and male buffaloes were more susceptible than female to HEV infection [42].

#### 2.1.3. Goats

Goats have been reported as a source of HEV infection in different countries. HEV circulates in goats and their products, such as milk [79,80,81,82,83]. HEV RNA was also detected in edible goat liver samples [81,83,84]. In Egyptian villages, goats usually reside in the same place as their owners and therefore human contact with these animals is high. To our knowledge, there are two reports about HEV in Egyptian goats. The first study was done in the Nile Delta and reported that 9.4% of the tested goats were positive for anti-HEV IgG and goats in this area are a potential source of zoonotic HEV infection in humans [42]. The second report was performed in villages of Assiut Governorate. Anti-HEV IgG and HEV Ag were recorded in 7.14% and 1.8% of goat milk samples, respectively [81]. Anti-HEV IgG and HEV Ag were also detected in the blood of goats with positive milk samples and the level of HEV markers was not different in both compartments [81]. HEV-positive goats were subclinical and there was no difference in liver transaminases between seropositive and seronegative goats. Besides, there was no evidence of extrahepatic replication of HEV in the goat kidney, as there were no HEV markers in the goat urine samples [81]. The isolated viruses from the goat belonged to HEV genotype 3, subtype 3a [81]. Since eating raw goat liver is common in rural communities, the authors tested the liver of five HEV seropositive goats for HEV RNA and two of them were positive. Interestingly, 80% of owners of seropositive goats were also seropositive to HEV, but they developed an asymptomatic infection and their liver function tests were normal [81].

#### 2.1.4. Sheep

Sheep are naturally exposed to HEV. HEV markers such as HEV RNA and anti-HEV antibodies were detected in the plasma and stool of sheep residing in different countries such as Italy, China, Spain, and Portugal [85,86,87,88]. Importantly, HEV is excreted in sheep milk [80] and a high risk of HEV exposure was recorded in shepherd workers and sheep milk cheesemakers [88]. HEV RNA was also detected in four (5.3%) out of 75 raw sheep liver samples in a Chinese slaughterhouse [86], where the butchers had a high HEV seroprevalence (57%), suggesting occupational exposure to the virus during animal contact [86]. Interestingly, one study showed that sheep were less exposed to HEV than goats [87]. The prevalence of anti-HEV antibodies was lower in sheep (2.8%) than in the goats (13.8%) present in the same farms [87]. A similar finding was recorded in Egypt; the lowest seroprevalence of HEV among Egyptian ruminants was recorded in sheep (4.4%) [42]. Although sheep are a common source of food for Egyptians, especially the Muslim community, there is no report on HEV in sheep and their products in Egypt.

### 2.2. Horse

HEV infection was reported in horses in several countries such as China, The Netherlands, Spain, the Republic of Korea, and Egypt [70,89,90,91,92,93]. Some studies showed that horses were positive for anti-HEV antibodies, but not for HEV RNA, with the seropositivity rate ranging from 12–18% of the tested animals [89,90,92]. Other studies confirmed the presence of HEV RNA in the horse samples and the isolated viruses belonged to HEV genotype 3 [70,91]. The isolated viruses from Spanish horses were closely related to the HEV strains circulating in humans and pigs [91]. Contact with horses was also a risk factor for increased HEV seropositivity among blood donors in Denmark [94]. Previous reports indicate a potential zoonotic HEV infection from horses.

In Egypt, Saad and colleagues assessed HEV markers and liver enzymes in workhorses in old Cairo, where horses were used to pull carts and were associated with the hide industry [93]. Among 200 horse sera samples, 26 (13%) samples tested positive for anti-HEV IgG [93]. Importantly, four out of 100 samples (4%) also tested positive for HEV RNA [93]. HEV RNA was recorded in only seronegative horses suggesting that the viremia is short-lived and terminated before the development of anti-HEV antibodies [93] Three isolates were 100% identical and closely related to the fourth isolate (99.6% similarity), indicating that the source of infection to these animals was the same [93]. Liver enzymes such as aspartate transaminases were only elevated in animals that tested positive for HEV RNA [93]. Surprisingly, the isolated strains belonged to HEV genotype 1 and were closely related to HEV strains isolated from humans in Egypt [17,93]. Importantly, HEV genotype 1 strains infect humans only and no animal reservoirs were confirmed for these isolates [7]. The previous findings could be explained by HEV crossing species and transmitting from humans to horses, which could be an accidental host for the virus. However, whether the virus can adapt and replicate inside the horse or not still needs further investigation.

### 2.3. Pigs

Pigs are well-known reservoirs for HEV [95]. HEV replicates in the liver and extrahepatic organs of pigs [96]. Therefore, edible products derived from pigs are a common source for HEV transmission [97,98]. Pig farms are present in Egypt and pigs are slaughtered in special abattoirs. Pig products such as sausages, meat and liver are commercially available in some local groceries/markets for non-Muslim residents. Studying HEV in pigs or their products in Egypt is very limited. To our knowledge, one study reported the presence of HEV genotype 3 in four pigs in an abattoir in Cairo [43]. The previous finding was reported almost 15 years ago and, since this time, no further studies were performed to confirm the association between pigs and HEV infection in Egypt. However, the number of pig abattoirs has been increasing in recent years in Egypt and recent studies have reported that pigs could be a source of infections for *Trichinella spiralis* and *Brucella suis* in humans [99,100].

### 2.4. Rodents

Rats are susceptible to rat HEV strains (HEV C1) [101,102]. Rat-derived HEV isolates are classified into *Rocahepevirus* genus (*Rocahepevirus ratti)* [4]. Recent studies confirmed that rat-derived HEV isolates cause infection in humans [23,24]. The clinical course of rat HEV infection in humans can be a mild self-limiting disease or can progress to fulminant hepatic failure [103]. In Egypt, Stoszek et al. reported that rodents could be potential sources of infection for Egyptians, especially in rural villages [43]. In a previous study, the authors reported that HEV was endemic in rural Egyptian villages and a high seroprevalence of HEV (67%) was reported among the villagers [43]. Follow-up on HEV-seronegative villagers showed that 3.7% became positive after 10.7 months [43]. Since most of these villagers were asymptomatic, the authors hypothesized that zoonotic HEV infection could be the cause of infection. Interestingly, it was mentioned that anti-HEV antibodies were reported in domestic animals and rodents in these same villages, suggesting that they could be the source of infection [43]. No further studies have been performed on Egyptian rodents to assess their roles in human HEV infection.

### 2.5. Cat

Kuno et al. reported a case of acute HEV infection in Japan which was associated with a pet cat [104]. The case’s family members were negative for HEV markers, while the cat was seropositive, suggesting a potential source of infection from a cat to a human [104]. In Germany, 21 out of 65 cats (32.3%) were HEV seropositive [105]. In The Netherlands, 14.89% of the tested cats were positive for anti-HEV antibodies [90]. In Italy, 3.1% of the tested household cats were HEV-seropositive [106]. Similarly, 5.4% of the tested cats in Turkey were positive for anti-HEV antibodies [107]. However, HEV RNA was not detected in the tested cats in several previous reports [90,105,106,107]. In Spain, a high seroprevalence (18.2%) was recorded in free-ranging and captive Iberian lynx (*Lynx pardinus*) and the captive cats were more seropositive than the free-ranging ones [108]. Interestingly, HEV RNA was detected in the stool of one cat and phylogenetic analysis revealed that the isolated strain belonged to HEV genotype 3, subtype 3f, with a high homology sequence identity with human HEV strains in this area [108]. In Egypt, no studies have been conducted on cats. However, one large study performed on 2428 Egyptian pregnant women showed that 84.3% of the women were positive for anti-HEV-antibodies, suggesting high endemicity of HEV in Egypt [55]. Importantly, one of the risk factors for HEV infection in these participants was frequent contact with cats [55].

## 3. Mode of HEV Transmission among Egyptians

There are four known routes of HEV transmission, fecal–oral, consumption of contaminated animal products (foodborne), transfusion-transmitted mode and from mother to baby. All these modes were reported among Egyptians

### 3.1. Waterborne Infection (Fecal–Oral Transmission)

Transmission of HEV through contaminated water is a common source of HEV infection in developing countries [109,110]. In this regard, HEV outbreaks are mainly associated with waterborne infection, where fecal contamination of the water system can spread the infection to many subjects and is associated with low sanitation in rural communities [7,111]. In Egypt, there was a waterborne HEV outbreak documented in the village of Kom El-Mansoura, Assiut Governorate, where HEV-infected cases and their household members or close contacts were using underground well water [54]. Though HEV was not isolated from the water in this outbreak, underground well water was the major risk factor reported [54]. In addition, the main risk factor reported by HEV-infected symptomatic patients or asymptomatic HEV seropositive subjects living in Egypt was the consumption of village water, public water, underground well water and/or municipal tap water [18,20,43,53,54,69]. Besides, HEV was detected by PCR in 27.7% of sewage samples derived from two wastewater treatment plants (WWTPs) in greater Cairo [112] and the virus was detected in the inlet, not the outlet, water source [112]. Importantly, 51% of the workers in these two studied plants were anti-HEV IgM positive, suggesting that the WWTPs were the source of infection [112]. Several studies reported coinfection of HEV with other hepatotropic pathogens transmitted via contaminated water such as HAV and schistosomiasis among Egyptians, suggesting a common route of infection [20,64,113].

### 3.2. Foodborne Transmission

In developed countries, HEV infection is a zoonosis that is transmitted by contact with HEV animal reservoirs, e.g., pigs, wild boar, deer, rabbits, etc, or consumption of their contaminated products [8,39,114]. Most infections are caused by HEV genotype 3 and 4 viruses [8,39,114]. Recently, ruminants and their products including milk have been reported as a potential source of HEV infection [74,83]. In Egypt, HEV markers were detected in cows’ and goats’ milk samples and the isolated viruses belonged to HEV genotype 3 [72,81]. In addition, HEV was detected in goat liver [81]. HEV genotype 3 was isolated from Egyptians [19,21]. In one case, the isolated virus from humans was related to the virus isolated from cow and goat milk samples and this patient was living in the same geographical region where the HEV-infected ruminants were present [21]. Among the risk factors of HEV infection among Egyptians is living in rural communities or villages where there was close contact with domestic animals. Taken together, these data suggest that zoonotic transmission is a potential mode of HEV infection in Egypt [21,43,55].

### 3.3. Blood Transfusion and HEV Transmission

Transfusion of contaminated blood or blood products such as RBCs, platelets, and plasma is a source of transfusion-transmitted (TT) HEV infection [115,116]. TT HEV infections are mainly caused by HEV genotype 3 [117,118]. Therefore, blood donors should be screened for HEV markers [117,119]. In Egypt, there is evidence of blood transfusion as a potential source of HEV infection. In this regard, Ibrahim et al. assessed the presence of anti-HEV IgM and HEV RNA among Egyptian blood donors. Three (0.45%) out of 760 random blood samples collected from healthy blood donors, distributed in 23 governorates, were positive for anti-HEV IgM. Two of these samples were positive for HEV RNA [120]. This finding suggests that, though low viremia of HEV was present among the Egyptian blood donors, blood transfusion could be a potential source of infection for Egyptians. A recent study was performed on 11,604 blood samples from apparently healthy blood donors in Qena Governorate in Upper Egypt [57]. In this study, higher HEV seroprevalence (28.7%) was reported among HCV and HBV seropositive blood donors [57]. The authors recommended screening of HEV among Egyptian blood donors [57]. Likewise, several studies confirmed a high prevalence of coinfection HEV/HBV and HEV/HCV among villagers, children, acute hepatitis patients and patients with chronic liver disease in Egypt [60,67,121] (Table 2). Since HCV and HBV are transmitted by blood transfusion, the high rate of coinfection of HEV with other blood-transmitted hepatotropic viruses suggests that blood transfusion is a potential route of transmission among Egyptians. Besides, anti-HEV antibodies (IgG, IgM) were detected among Egyptian children who were associated with transfusion-dependent thalassemia [66] and there was a significant correlation between HEV seropositivity and the amount of blood transfused per year [66].

### 3.4. Vertical Transmission

HEV is transmitted from infected mothers to their offspring [27]. The outcomes of vertical HEV transmission depend mainly on the viral genotype and the trimester of pregnancy. HEV genotype 3 causes mild self-limiting infection among pregnant women [122,123], while HEV genotype 1 causes severe outcomes and death for mothers and babies, especially in the third trimester of pregnancy [124]. In Egypt, 70–84% of the pregnant women in rural communities were HEV seropositive [55,65]. Most were asymptomatic and the infections were subclinical [18,53]. On the other hand, El-Esnawy reported that HEV infection caused abortion among Egyptian women. HEV RNA and HEV Ag were reported in 16% and 20% of the aborted women, respectively [125]. Importantly, HEV Ag was recorded in 5% of fetal tissues, suggesting vertical HEV transmission and its adverse complications [125].

## 4. Hepatitis E Virus Is a Neglected Disease in Egypt despite the High Seropositivity Reported among Egyptians

In the past two-three decades, several studies have been conducted on Egyptians to assess the seroprevalence of HEV and risk factors of infection. The main finding in several reports was that HEV infection is endemic in Egypt, especially in villages and rural communities [43,55,62,63]. The seroprevalence of HEV among Egyptians was the highest in the world and can reach up to 84% [55,62]. The high seroprevalence among females and children during the first decade suggested that all Egyptians were probably exposed to HEV during childhood [55,65,68]. A follow-up study was performed on Egyptian villagers from rural communities and reported an incidence rate of 41.6/1000 persons per year of HEV infection [43]. Table 1 summarizes the incidence and seroprevalence of HEV among Egyptians in different governates.

### 4.1. Risk Factors of HEV Exposure for Egyptians

Studies showed that several factors are associated with HEV exposure and infection among Egyptians. Residence in rural communities and villages is the main risk factor for HEV infection (Table 1). Poor socioeconomic conditions, low hygiene and crowded homes are common in these communities. Besides, animals, especially ruminants, are living in the same home or close to humans. Direct contact with animals is common, animals defecate close to humans, and this becomes a source of water contamination. Using underground water and public water sources were always reported among the risk factors for HEV exposure. Food-borne infection is another risk factor of HEV transmission to Egyptians, especially through ingestion of ruminant milk and liver [21,72,81]. Due to the crowdedness of people in Egyptian villages, the transmission of HEV infection to family members or close contacts was reported [53,54]. Age, pregnancy, coinfection with other hepatotropic pathogens, leukemia and blood transfusion were other risk factors for HEV infection among Egyptians [21,57,126].

### 4.2. Coinfection of HEV with Other Endemic Hepatotropic Pathogens in Egypt (Table 2)

HEV was reported in coinfection with other hepatotropic pathogens which were endemic in Egypt. HEV was a coinfection among patients infected with bloodborne viral hepatitis such as HBV and HCV and enterically- transmitted pathogens such as HAV and *Schistosoma*. HCV and HBV were endemic in Egypt and the highest prevalence of HCV globally was reported in Egypt [66,127], although it has been recently declining due to the treatment of infected subjects with direct acting antivirals [128]. HEV/HCV coinfection was reported among different subjects, such as blood donors, pregnant women, villagers, and acute hepatitis patients [60,65,67]. HEV/HCV coinfection resulted in a worse prognosis compared to monoinfection [65,67]. Similarly, HEV was detected in HBV-infected children and adults [60,121]. The previous findings suggest that blood transfusion could be a source of HEV infection among Egyptians. Coinfection with HEV/HAV was also reported in children [121]. Schistosomiasis alters the immune system and was reported as a risk factor for HEV infection, especially among Egyptian villagers [113].

### 4.3. Waterborne Outbreaks Reported in Egypt

During 2007–2008, 235 acute viral hepatitis patients were admitted to Assiut University Hospitals. Acute HEV infection was confirmed in 42 patients. Importantly, 14 patients were living in one village, Kom El-Mansoura [54]. Screening of family members and close contacts with acute HEV patients living in the same village revealed that the other 14 members were positive for anti-HEV IgM, though the infection was subclinical [54]. The major risk factor reported by the patients and close contacts was the use of underground water, hypothesizing that the outbreak was waterborne in nature [54]. However, the virus could not be isolated/detected in the water a few months after the outbreak, indicating either a short-lived infection or the possibility of another source of infection [54].

### 4.4. HEV Detection Is Neglected in Egyptian Hospitals

Despite the above-mentioned data and the spread of HEV infection in different governorates in Egypt (Table 1 and Table 2), the diagnosis of HEV is not regularly performed in Egypt. Therefore, the actual estimation of HEV among hospitalized patients is not known and HEV is neglected and underestimated. The neglect of this pathogen, despite its endemicity in Egypt, could be attributed to the following: (A) Reports showed that most HEV infections were subclinical and asymptomatic, and most Egyptians acquired the disease during childhood [43,55]. (B) The reported course of HEV infection among acute viral hepatitis cases was a self-limiting disease with no severe complications even during the outbreaks and pregnancy, giving an early indication that the circulating HEV isolates in Egypt were avirulent or attenuated [18,26,53,54]. (C) The characterized HEV strains were limited in Egypt. In most situations, the virus could not be isolated from the expected sources of infection such as water, animal, etc., suggesting a hypothesis that the infection was transient, or the source of infection was not completely known [18,54]. Therefore, the diagnosis of viral hepatitis in Egyptian hospitals is focused mainly on the identification of viruses that cause more severe complications, such as HBV, HCV and HAV.

### 4.5. Recent Findings on the Status of HEV Infection in Egypt

In the past six years, some studies had been conducted on HEV in Egypt that showed important findings which should be taken into consideration. An initial screening of healthy blood donors showed a low level of viremia and HEV incidence among Egyptian blood donors [120]. On the other hand, a recent study performed on a larger cohort of blood donors showed that a total of anti-HEV antibodies were detected in 193 samples of HCV/HBV seropositive blood samples, indicating the increase in the incidence of HEV markers among blood donors [57]. Sayed et al. also reported that 10% of acute hepatitis of unknown etiology (AHUE) samples were positive for HEV markers [21]. Importantly, four cases progressed to severe complications (fulminant hepatitis) and died [21]. Another cohort reported by El-Mokhtar and colleagues showed fulminant hepatitis and death in patients with acute HEV infections [56]. The previous studies alerted researchers to the complications of HEV infection among Egyptians [21,56]. Similarly, earlier studies reported that ruminants and domestic animals were HEV seropositive, but there was no report on HEV isolation or characterization in these animals. Recent studies confirmed the presence of HEV-3 in ruminants’ edible products such as milk and liver samples [72,81]. These results confirm the previous hypothesis about zoonotic HEV transmission in Egypt [72,81,129]. HEV was also misdiagnosed as drug induced liver injury (DILI) cases among Egyptians. Out of 80 DILI samples, 12 (15%) were HEV infections suggesting the unmet need for HEV diagnosis in DILI in Egypt [130]. In a parallel line, several studies showed the necessity of testing HEV markers before diagnosing cases as DILI [131,132]. Besides, the detection of HEV in patients’ PBMCs and urine, but not semen, indicates that the viral isolates circulating in Egypt mediate extrahepatic manifestations [22,133].

## 5. Summary, Conclusions and Future Perspectives

The initial view of HEV infection in Egypt has been changing, from it being a subclinical infection caused by attenuated isolates to a symptomatic disease with the possibility of progression to fulminant hepatic failure. In addition, contaminated water caused by low sanitation practices is not the only source of HEV infection in Egypt and zoonotic HEV infection is becoming another possible source. These changes suggest either modifications in this neglected pathogen over time or emerging of new isolates that cause the current changes. Unfortunately, we do not know many details about the old strains circulating in Egypt. Therefore, we could not compare the new strains and the old.

Research in the HEV field is growing rapidly and therefore more facts are coming to light. Importantly, the advances in molecular and serological approaches help in improving the detection and diagnosis of HEV. However, in the past, many facts about HEV were not known, misinterpreted and/or misunderstood due to the absence of these recent approaches. This point again raises the same question: are the changes seen in HEV status in Egypt due to emerging new isolates or merely modifications of the old, neglected ones?

It is becoming clear that diagnosis of HEV should be included in AHUE and blood donors’ screening. This process will help to reduce the incidence of HEV infections among Egyptians and will make clinicians aware of the actual status of HEV in Egypt. Besides, clinicians can recommend anti-HEV therapies to prevent the progress to fulminant hepatitis. There are no specific therapies for HEV. However, ribavirin is used off-label in chronic HEV infection [134], though its use is restricted in other situations such as pregnancy. Previous studies showed that ribavirin has a good effect in acute severe cases and could prevent complications [135]. In parallel, ribavirin or other HEV-specific therapies could reduce the mortality associated with HEV-mediated fulminant hepatic failure, if the diagnosis of HEV could be accomplished early.

HEV is a zoonotic disease and the risk of HEV infection is increasing by contact with animals and/or consumption of contaminated animal products. In Muslim counties such as Egypt, pigs are less likely to be the main reservoir for HEV. However, in Israel, HEV genotype 3 subtype 3f was detected in pigs, though there was no HEV-3 infection among humans [12]. Consumption of pig products was not likely to be the main source of infection, therefore HEV infection in Israel is still questionable [12]. However, ruminants are common in Egypt, especially in rural communities, and could be a reservoir and a source of infections there. Frequent testing of ruminants for HEV and monitoring them over time becomes crucial. Screening of milk products for HEV and adequate cooking of ruminant meat/liver could reduce the risk of zoonotic HEV infections. It is also important to expand the screening of other potential HEV reservoirs in Egypt such as rodents, camels, pigs, etc.

## Figures and Tables

**Table 1:** The prevalence of HEV among Egyptians distributed in different governorates and the risk factors for infection.

Governorate or Region	Study Subject	HEV Markers Tested	Main Findings	Risk Factors	Reference
Cairo	Acute hepatitis children (n = 261)	Anti-HEV IgM by western blot	anti-HEV IgM was detected in 22% of children	Living in home not connected to a municipal source of water	[58]
	Acute hepatitis adults (n = 219), from whom 143 patients were included in analysis	Anti-HEV IgM and IgG	HEV was detected in 21.7% of samples. HEV prevalence was higher than HAV, and HCV.	Age and rural residents	[59]
Greater Cairo (Cairo, Giza and, El Qalyoubia)	Acute hepatitis patients (n = 1950)	Anti-HEV antibodies (IgM and IgG) HEV RNA	Acute HEV infection was recorded in 17 cases. Coinfection with other hepatotropic viruses HEV genotype 1 is the circulating virus	Rural residence Contact with animals unsanitary toilets	[60]
Nile Delta	Residents of villages (healthy subjects, n= 155)	anti-HEV antibodies	57% HEV seroprevalence among children 4–9 years. The rate of HEV seroprevalence did not increase with old age HEV in endemic in villages	Village, low sanitation	[61]
	Pregnant women (n= 2428) in the second and third trimesters from three villages	anti-HEV antibodies	Anti-HEV prevalence was 84.3%. No history of liver disease Asymptomatic Almost all Egyptian women exposed to HEV during the childhood.	Contact with cats Not using soap Old age Many siblings	[55]
Nile Delta + Upper Egypt (Initial study)	Villagers from two Egyptian rural communities (a) Nile Delta: participants (n= 3997) represented 35.7% of the inhabitants (b) Upper Egypt: participants (n= 6029) represented 55% of inhabitants	anti-HEV antibodies	The prevalence of anti-HEV antibodies was among the highest reported globally. Anti-HEV prevalence > 60% in the first decade of life and from the third decade to the eight decades Anti-HEV prevalence is peaked (76%) in the second decade	Village, low sanitation, rural communities, rural habits.	[62]
Nile Delta + Upper Egypt Follow up Study	Follow up study was done 10.7 later months on villagers (n = 2411) who participated in the study [62]	anti-HEV antibodies	3.7% of the villagers who tested negative at the initial study became HEV seropositive. The incidence of anti-HEV was estimated to be 41.6/1000 person-years. HEV is highly endemic and asymptomatic subjects	Use of municipal tap water for drinking. Food from street vendors Zoonotic (anti-HEV antibodies were detected in domestic animals and rodents in the same villages	[43]
Nile Delta (Mansoura Governate) + Upper Egypt (Asyut Governate)	Acute hepatitis patients (n= 287)	Anti-HEV IgM, IgG, HEV RNA	Acute HEV markers was recorded in 20.2%. HEV RNA was isolated from stool of two patients. The isolated viruses belonged to genotype 1 and were infectious to non-human primates.	Use of public water (outside the residence) Use of well water	[18]
Nile Delta Kafr El Sheikh Governate	1850 villagers representing 68% of the total population	Anti-HEV IgG	The seroprevalence of anti-HEV was 17.2%. HEV was endemic in the rural regions	Village, rural communities, poor Socioeconomic and sanitary practices Anti-HEV seroprevalence increased by age	[63]
	patients who had a history of jaundice and hospitalized (n = 134) ruminant present in the same geographic area (n = 185, cows n = 51, buffaloes n = 57, sheep n = 45 and goat n = 32)	anti-HEV IgG antibodies	38% of the patients were HEV seropositive. Anti-HEV IgG was detected in 21.6%, 14%, 4.4% and 9.4% from examined cows, buffaloes, sheep and goats, respectively.	Zoonotic transmission through ruminants Rural communities Food borne infection	[42]
Nile Delta Dakahlia Governate (Mansoura)	Acute hepatitis children (n = 180)	Anti-HEV IgM, IgG HEV RNA.	HEV was recorded in 26.1% of the patients	Rural residence Presence of autoimmune antibodies Coinfection with HAV. Contact with animals	[64]
	Asymptomatic pregnant women (n = 116)Two groups: Group1: HCV seropositive (n-56) Group2: HCV seronegative (n-60)	Anti-HEV IgG	Significant higher HEV seropositivity in HCV seropositive women (71.42%) than HCV seronegative women (46.7%). HEV/HCV coinfection caused worsening of the biochemical liver indices.	Rural residence Chronic HCV infection	[65]
	β-thalassemia children (n = 140)	Anti-HEV IgM and IgG	24.29% of patients were anti-HEV IgG positive and 2.86% were anti-HEV IgM positive.	Blood transfusion Amount of blood transfused Rural residence	[66]
Nile Delta Gharbia Governorate	village residents n = 2085, from which 505 were active HCV-infected patients	Anti-HEV IgG	Anti-HEV IgG was detected 71.4% of villagers with chronic HCV. Anti-HEV IgG was detected 96.1% with advanced liver disease (cirrhosis and hepatocellular carcinoma). No anti-HEV IgM	Villages and rural communities’ habits Chronic liver disease HCV	[67]
Alexandria	healthy adolescent females (n = 95), from whom 10 were pregnant.	Anti-HEV antibodies	The prevalence rate of anti-HEV antibodies was 38.9%. No HEV in pregnant Subclinical infection HEV in endemic in Alexandria	Anti-HEV seroprevalence increased by age Education	[68]
	Acute hepatitis patients (n = 202 samples)	Anti-HEV IgM, IgG HEV RNA.	Anti- HEV IgG was detected in 44.5% of patients Anti-HEV IgM was detected in 24.2% of patients HEV RNA was detected in 5.3% of stool samples The isolated virus belonged to HEV genotype 1 and closely related to Burma strain (subtype 1a)	Residents of rural area Use of common village water Taking oral therapy of schistosomiasis Use of indoor dry pit	[20]
Asyut Governate	Acute hepatitis patients (n = 235) & asymptomatic contacts (n = 200)	Anti-HEV IgM and anti-HEV IgG	HEV was detected in 16% of acute hepatitis patients and 7% of asymptomatic contacts.	Residents in rural area. Use of underground water Contact with animals	[53]
	Acute hepatitis children (n = 123)	Anti-HEV IgM and anti-HEV IgG	HEV was detected in 30.9% of the patients	Drinking of contaminated water Use of underground water	[69]
	Acute hepatitis of unknown etiology (AHUE), Hepatitis of non-A-C (n = 300)	Anti-HEV IgM, anti-HEV IgG HEV RNA.	10% of AHUE patients was active HEV infection The isolated viruses were genotype 1 subtype 1b and 1e except one case was genotype 3 subtype 3a Acute HEV cases were symptomatic and self-limiting disease and 4 cases progressed to fulminant hepatic failure	Old age Leukemia History of preexisting liver disease Drinking ruminants’ milk Rural residence	[21]

**Table 2:** Co-infection of HEV with other hepatotropic pathogens in Egypt.

Coinfection	HEV Markers Tested	Findings	Region	Reference
HEV/HAV	Anti-HEV IgM and IgG	A study conducted on 162 children. Seroprevalence of IgM and IgG antibodies were detected in 4.5% and 34.1% of acute HAV infected children, respectively.	Dakahlya Governorate (Mansoura)	[121]
HEV/HBV	Anti-HEV IgM and IgG	A study conducted on 162 children. Anti-HEV IgM and IgG were detected in 3.3% and 56.7% of HBV infected children, respectively.	Dakahlya Governorate (Mansoura)	[121]
	Anti-HEV antibodies (IgM and IgG) & HEV RNA	HEV was detected in two out of 771 HBV infected patients.	Greater Cairo	[60]
	Total anti-HEV antibodies	HEV (n = 102) was documented in blood donors tested positive for HBV (n = 295)	Qena Governorate, Upper Egypt	[57]
HEV/HCV	Anti-HEV antibodies (IgM and IgG) & HEV RNA	HEV was detected in one case of acute HCV infected patient (n = 318)	Greater Cairo	[60]
	Anti-HEV IgM and IgG	A study conducted on 162 children. Anti-HEV IgG was reported in 52% of HCV infected children.	Dakahlya Governorate (Mansoura)	[121]
	Anti-HEV IgG	Anti-HEV IgG was detected in 411 out of 505 active HCV-infected villagers. HCV-HEV co-infection cause a worse prognosis. Anti-HEV IgM was not detectable in HCV-infected villagers.	Gharbia Governorate, Basyoun, Nagreej village	[67]
	Anti-HEV IgG	Anti-HEV IgG was detected in 40 (71.4%) out of 56 HCV-seropositive pregnant women. HCV-HEV co-infection caused a worse liver biochemical index.	Dakahlya Governorate	[65]
	Total anti-HEV antibodies	HEV was positive in 88 blood donors who tested positive for HCV (n = 370)	Qena Governorate, Upper Egypt	[57]
HEV/HBV/HCV	Total anti-HEV antibodies	HEV was detected in three blood donors who tested positive for both HCV and HBV (n = 6)	Qena Governorate, Upper Egypt	[57]
	Anti-HEV IgM and IgG	A study conducted on 162 children. Anti-HEV IgG was reported in 30% of HBV/HCV infected children.	Dakahlya Governorate (Mansoura)	[121]
	Anti-HEV antibodies (IgM and IgG) & HEV RNA	Acute HEV was detected in acute hepatitis patient coinfected with HCV/HBV	Greater Cairo	[60]
HEV/*S. mansoni*	Anti-HEV IgM	Anti HEV IgM was assessed in *S. mansoni* infected patients (n = 100) and control subjects (n = 100). Anti-HEV IgM was higher in *S. mansoni* infected patients (31%). Schistosomiasis is a risk factor for HEV infection by altering the immune system	Rural communities	[113]

## Data Availability

Not applicable.
